# Biocomposite Scaffolds for Tissue Engineering: Materials, Fabrication Techniques and Future Directions

**DOI:** 10.3390/ma17225577

**Published:** 2024-11-15

**Authors:** Naznin Sultana, Anisa Cole, Francine Strachan

**Affiliations:** The Texas Undergraduate Medical Academy, Prairie View A&M University, Texas A&M University System, Prairie View, TX 77446, USA; acole24@pvamu.edu (A.C.); fstrachan@pvamu.edu (F.S.)

**Keywords:** tissue engineering, biopolymers, chitosan, gelatin, collagen, pectin, fabrication, techniques, applications, lyophilization

## Abstract

Tissue engineering is an interdisciplinary field that combines materials, methods, and biological molecules to engineer newly formed tissues to replace or restore functional organs. Biomaterials-based scaffolds play a crucial role in developing new tissue by interacting with human cells. Tissue engineering scaffolds with ideal characteristics, namely, nontoxicity, biodegradability, and appropriate mechanical and surface properties, are vital for tissue regeneration applications. However, current biocomposite scaffolds face significant limitations, particularly in achieving structural durability, controlled degradation rates, and effective cellular integration. These qualities are essential for maintaining long-term functionality in vivo. Although commonly utilized biomaterials can provide physical and chemical properties needed for tissue regeneration, inadequate biomimetic properties, as well as insufficient interactions of cells-scaffolds interaction, still need to be improved for the application of tissue engineering in vivo. It is impossible to achieve some essential features using a single material, so combining two or more materials may accomplish the requirements. In order to achieve a proper scaffold design, a suitable fabrication technique and combination of biomaterials with controlled micro or nanostructures are needed to achieve the proper biological responses. This review emphasizes advancements in scaffold durability, biocompatibility, and cellular responsiveness. It focuses on natural and synthetic polymer combinations and innovative fabrication techniques. Developing stimulus-responsive 3D scaffolds is critical, as these scaffolds enhance cell adhesion and promote functional tissue formation while maintaining structural integrity over time. This review also highlights the natural polymers, smart materials, and recent advanced techniques currently used to create emerging scaffolds for tissue regeneration applications.

## 1. Introduction

The study of tissue engineering (TE) scaffold is an important field that researchers have studied to determine if it is a less invasive and effective treatment for regenerative medicine. The cellular process of the TE scaffold technique involves inserting a non-toxic component into the body, known as the scaffold, through which the cells can attach, migrate, differentiate, and proliferate. Biocompatible materials that mimic the cellular structure and function of diseased or damaged tissues have been widely studied in tissue engineering. Secretion of the natural extracellular matrix (ECM) takes place to regenerate the damaged tissue when the surrounding tissue absorbs the biocompatible materials. Most cell types can attach to the ECM, and this event aims to fabricate TE scaffolds from biodegradable and biocompatible polymers. Currently, there are obstacles to using biological substitutes to form tissue scaffolds. For this reason, it is essential to modify and fabricate an optimal tissue scaffold that is non-toxic and does not interfere with the immune system or cellular transporting mechanisms. When properly fabricated, biodegradable components have the potential to replace the ECM temporarily and have properties that can induce the biomolecule and/or drug release for anti-inflammatory and antibacterial activities in the tissue for regeneration.

Tissues and organs play an essential role in organisms, and any damage to the tissues impairs the body’s normal functioning and the quality of life of an organism. Although tissues such as bones and skeletal muscles can heal themselves after incurring some form of injury, clinical interventions are required to address severe injuries [1]. Under the circumstances, conventional treatment methods, including autografts or donor grafts, may not work effectively because they take longer to recover, have an increased risk of infection, or may be immunologically rejected [2]. As a result, TE is the most effective alternative to guarantee a patient’s full recovery in the case of severe tissue damage [3]. TE restores, improves and maintains tissues that have been damaged due to injury or disability that arises from congenital conditions. Successful tissue regeneration is achieved using natural and synthetic polymers.

Developing biocomposite scaffolds with appropriate properties is very important in TE. Regenerative medicine is an interdisciplinary field that applies life sciences and engineering principles to promote regeneration or restoration of injured tissues and organs. Using regenerative medicine, tissues, and organs damaged through aging, trauma, or disease can be healed or even replaced [4]. Central to regenerative medicine is TE. TE is a field in which biological and engineering principles are employed to create new tissues and organs [5]. TE is required because it provides an alternative source of biocompatible and biodegradable tissues and organs, which addresses the crucial gap where there is a high demand for tissues and organs but a limited supply.

**Figure 1:**
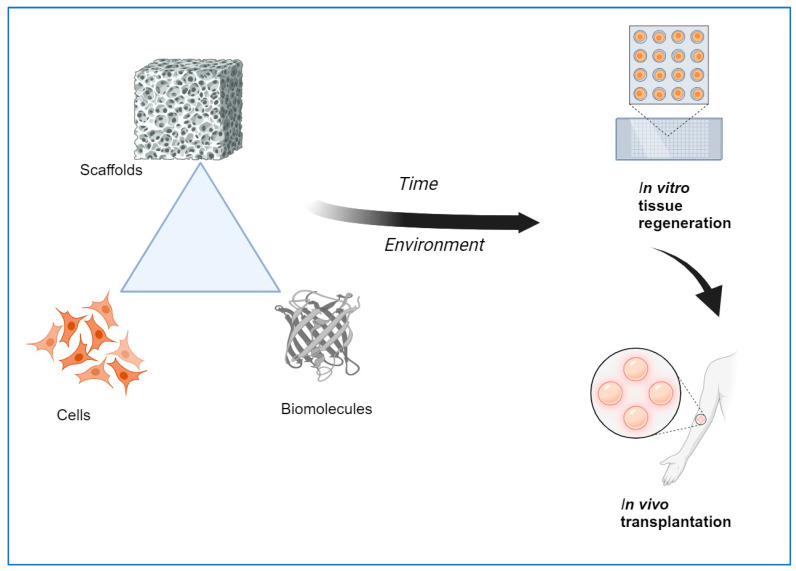
Schematic diagram of Tissue Engineering Construct (Created with BioRender.com).

For TE to be functional, a strategy of regenerating tissues and organs, a triad of cells, scaffolds, and growth factors are usually employed (Figure 1). The goal is to either stimulate the original tissue to regenerate it, or to replace the original tissue. A scaffold is used not only to provide a structure similar to that provided by an extracellular matrix but also to provide a framework where biological cues can be used, added, or infused to promote regeneration and mimic an environment found naturally in healthy tissues and organs.

The use of scaffolds in TE has evolved throughout history. Despite being constructed from varying materials, such as linen in Egypt, catgut in Europe, and ant heads in India and South Africa, these scaffolds provided a framework for a tissue to heal or regenerate. One common barrier faced by many pioneers in scaffold creation was constructing scaffolds that could not only provide a structural framework but also facilitate cell attachment, differentiation, and proliferation. The ideal scaffold must also allow for the crucial cell-to-cell and cell-to-matrix interactions demonstrated in vivo [6].

Scaffolds in TE are three-dimensional and porous, providing a favorable environment for tissue reparation and regeneration [7]. There are several requirements for scaffolds. The ideal scaffold should be biodegradable, biocompatible, possess mechanical properties, have a highly porous architecture, and be cost-effective. Cell adhesion and proliferation rely on the scaffold’s biocompatibility. Furthermore, another measure of how biocompatible a scaffold can be is an immune response. A negligible immune response is ideal for preventing chronic inflammatory reactions that impede healing or may even cause rejection [7].

This review comprehensively analyzes various biomaterials used to fabricate biocomposite scaffolds used in TE, focusing on their materials, fabrication techniques, and future directions. It also aims to synthesize current knowledge on scaffold properties and their impact on tissue regeneration. By examining various materials and methods, the review seeks to identify the strengths and limitations of each approach, offering insights into potential advancements in scaffold technology. The scope of the review spans from foundational principles to the latest innovations to ensure a thorough understanding of the field’s current state and future possibilities.

## 2. Natural, Synthetic, and Composite Materials Are Used to Fabricate TE Scaffolds

Determining material properties such as chemical, mechanical, and structural properties are essential to ensure that the fabricated scaffold can resist dynamic remodeling during in vivo processes. Criteria such as scaffold biocompatibility are essential to reduce the risk of scaffold rejection with minimal immune response in the body. The properties of an ideal scaffold that can closely mimic the natural bone should be biodegradable, mechanically stable, biocompatible, and highly porous morphology. To fabricate scaffolds for bone or skin regeneration, biomaterials such as biodegradable polymers are widely used as the material of scaffolds. Using biodegradable polymer as a scaffold will provide adequate support for cells and degrade at a rate that coincides with tissue growth. Biodegradable polymers are divided into biodegradable synthetic polymers and natural polymers. Synthetic polymers such as poly(lactic acid) (PLA), poly(glycolic acid) (PGA), and poly(caprolactone) (PCL) are widely used as polymer materials for biodegradable scaffolds. The typical mixing of different materials is a conventional method of producing the scaffold. By choosing materials with different characteristics that can complement one another, the properties of the scaffold, such as surface wettability, degradation, mechanical properties, and cell affinities, can be manipulated according to preference. Consequently, hydroxyapatite (HA) can be mixed with synthetic biopolymers to produce composite materials for bone TE scaffolds, as HA is a principal constituent of natural bone. This incorporation will render the scaffold with biological responses and mechanical properties that closely mimic the natural bone, especially when incorporating the nanoparticle hydroxyapatite (nHA) with a nanofibrous polymer matrix.

However, cell affinity towards synthetic polymers is usually poorer than natural polymers. PCL has the advantages of being biodegradable and biocompatible, but it has lower hydrophilicity, slower degradation rate, and lack of surface cell recognition sites. On the other hand, gelatin is a natural polymer derived from collagen, the main structural component of the ECM of skin. It is hydrophilic and has a faster degradation rate. Due to the merit of its biological origin, it can be blended with PCL to achieve a desired fibrous scaffold, which mimics ECM with enhanced wettability, flexibility, and faster degradation rate and promotes cell attachment and proliferation.

The primary natural polymers in TE scaffolds include chitosan, collagen, and chitin. Collagen forms part of the extracellular matrix in most connective tissues within the body of mammals. Generally, it is composed of fibrous protein [8]. Since it has a fibrillar structure, collagen plays an essential role in maintaining the biological structure and integrity of the extracellular matrix, thus providing support for tissues [9]. Furthermore, collagen is less immunogenic, more permeable, biocompatible, and biodegradable, and has a structural porosity. Unfortunately, collagen does not have the mechanical strength ideal for complex tissue engineering [10]. As a result, bioceramics, such as hydroxyapatite (HA) and beta-tricalcium phosphate (β-TCP), are used with collagen to fabricate composite scaffolds to improve strength [10].

Furthermore, β-TCP is essential in providing better osteoconductivity and accelerating the scaffold degradation rate for replacement with a newly formed tissue [10].

Gelatin used in tissue engineering scaffolds is mainly a protein derived from collagen through hydrolysis [11]. Since it contains arginyl glycyl aspartic acid (RGD), gelatin is highly biocompatible. Furthermore, the arginyl glycyl aspartic acid found within the gelatin structure makes it easy for cells to attach, spread, and proliferate [12]. Since gelatin has poor mechanical properties, it cannot be directly used to treat bone defects. Instead, gelatin-based scaffolds are combined with other materials, including silica nanoparticles and polymer microparticles, to improve their mechanical strength [12].

Another important natural polymer employed in tissue engineering scaffolds is chitosan. Chitosan is made up of a linear polysaccharide, usually found in crustaceans, mollusks, and the cuticles of insects [13]. It is biologically compatible, degradable, adhesive, and renewable [14]. Furthermore, it contains free amino groups, which can be involved in protonation, thus making chitosan easy to modify using biochemical groups [13]. The protonation of amino groups makes it easy for chitosan to electrostatically interact with DNA, proteins, lipids, or any other negatively charged synthetic polymers [15]. Mechanical enhancers are used together with chitosan to improve its mechanical strength. Table 1 shows the natural polymers commonly used to fabricate tissue engineering scaffolds.

Recently, another natural polymer, pectin, developed with chitosan, was explored in tissue engineering as a scaffolding material to regenerate bone and skin tissues (Table 2). Blending chitosan and pectin produces a polyelectrolyte complex that outcomes a new scaffold that has enhanced mechanical resistance, swelling capacity, porous microstructures, stabilized crosslinking, and biocompatibility [19].

Biodegradable polymer matrix nanocomposites are highly effective for bone tissue engineering [20]. These materials have regenerative capabilities as they were shown to stimulate the regeneration of bones and tissues. Surav et al. emphasized natural biopolymers because they are cost-effective, have high availability, cause low immune response, have negligible toxicity, and are biocompatible. Natural biopolymers such as Hyaluronic acid, elastin, alginate, collagen, gelatin, chitosan, and GAGs (glycosaminoglycans) are some of the natural polymers used in scaffolds for bone tissue engineering, and they are also the most researched [20].

Conversely, synthetic scaffolds meet the inconsistency found in natural scaffolds, which tend to differ from batch to batch. Furthermore, the same ethical concerns about animal-derived scaffolds do not apply to synthetic ones. With synthetic scaffolds, there is room for modification, higher availability, and low costs. Polyglycolic acid (PGA) and polylactic acid (PLA) have been investigated extensively in bone tissue engineering [21]. After implantation, PGA scaffolds, made from a material with high crystallinity, also demonstrated controlled degradation with decreased mechanical strength. Osteoblasts have been shown to proliferate and calcify on PGA scaffolds. PLA scaffolds are known for their mechanical and thermal properties. In addition to being biocompatible, pure PGA scaffolds also allow for the expression of osteogenic bone markers and the formation of calcium nodules [21].

Poly(caprolactone) (PCL) is a semicrystalline biocompatible synthetic polymer. Due to its biocompatibility, superior rheological properties, and elasticity, PCL polymer has been fabricated into many forms, such as films, matrices, membranes, micro/nanoparticles, capsules, fibers, and reservoir devices for drug delivery purposes and tissue engineering. PCL has relatively low stiffness, limited cell affinity, and is hydrophobic by nature, thus restricting its range of clinical applications for bone tissue regeneration. One of the strategies to overcome this limitation is to combine PCL polymer with particles that have bioactive effects, such as nHA, biphasic calcium phosphate (BCP), and bioactive glasses [22]. Since an adequate hydrophilic surface is essential for cell attachment and to control biological interactions between the materials, different surface modification techniques have been applied to the PCL fibers, such as plasma treatment, chemical treatment with sodium hydroxide, coating or adsorbing natural ECM proteins, and blending with biologically active molecules.

In plasma treatment, oxygen-containing groups such as gases (air, O_2_, NH_3_, SO_2_, CO_2_), organic compounds, or polarized groups (hydroxyl, carboxyl, amino, and sulfate) are introduced on the fiber’s surface. A previous study reported the influence on the biological interactions of the fiber after undergoing plasma treatment. Prabhakaran et al. observed that the PCL fibers exhibited higher proliferation of Schwann cells than the PCL and PCL–collagen fibers without the treatment [23]. As for the chemical treatment technique, sodium hydroxide (NaOH) is commonly used on PCL fibers. The carboxylate and hydroxyl groups are introduced to improve the wettability of PCL.

Another surface modification technique practiced for PCL fibers is the coating using protein adsorption to promote cell interaction. Protein, laminin, gelatin, collagen vitronectin, and fibronectin can provide signaling cues and adhesion ligands for cell functions. Koh et al. used laminin to promote neurite outgrowth and adsorbed onto the surface of PCL fibers. Lastly, the blending technique of surface modification for PCL using biologically active materials has been reported to compensate for the lack of several biological properties of PCL, such as low mechanical properties and bioactivity.

Composite scaffolds contain a combination of biomaterials: biopolymers, bioceramics, degradable metals, or natural products. When creating scaffolds for TE naturally, a composite material, such as bone, is needed, and it is logical to utilize a composite scaffold for increased biomimicry and bioactivity [22]. Many materials are used in these composite scaffolds; Turnbull et al. outline polymers, hydrogels, metals, ceramics, and bio-glasses, to name a few [24]. These composite scaffolds demonstrate desirable mechanical properties and cell interactions.

Several properties, including the poor mechanical properties, hydrophilicity, and conductivity of biopolymers, can be improved by combining them with conductive polymers in the biocomposite mixture. Conductive polymers (CP) show a conjugated structure consisting of alternating carbon–carbon double bonds. Due to their appropriate biocompatibility, CPs are used in different biomedical applications, including TE applications. These materials can mediate electrical stimulation, an important stimulating factor for cell activity. The most crucial property of conducting polymers is their electrical conductivity, so the first approach is to study their electrical-related biological behaviors. Neurons are well known for the membrane-potential-wave style signal transduction. Therefore, the study of CPs first focused on the electrical stimulation of neuron cells. In addition, electrically active tissues (such as the brain, heart, and skeletal muscle) provide opportunities to couple electronic devices and computers with human or animal tissues to create therapeutic body–machine interfaces. The piezoelectric property makes an endogenous electrical field due to strains that alter cell proliferation. It was reported that such stimulations improve osteoblast activities, including proliferation and adhesion.

Furthermore, previous studies indicated that adding CP can enhance the biodegradability and mechanical strength of the matrix and its proper biocompatibility properties. The most significant drawback of CP for scaffold application is their degradability in the natural environment, which may cause chronic inflammation. Therefore, a combination of this polymer with biodegradable ones could eliminate the problem [25,26]. The most frequently used CPs for TE applications include polypyrrole (PPy), PEDOT: PSS, and polyaniline (PANi). It was reported that bio nanocomposite conductive TE scaffolds fabricated by incorporating conductive PEDOT: PSS into nanohydroxyapatite/chitosan using a lyophilization technique exhibited notably higher cell attachment [25]. Electrospun membranes constructed by dipping PLA/PHBV into conductive PEDOT: PSS solution showed higher wettability and improved surface roughness, which caused higher cell attachment, cell viability, and proliferation compared to their uncoated counterparts [26].

Several applications have been reported for polypyrrole (PPy) in tissue engineering and drug delivery. One of the studies was carried out on a polycaprolactone fumarate–polypyrrole (PCLF–PPy) based scaffold for application on conductive nerve conduits [27]. The PC12 cell line was cultured on the prepared nanocomposite scaffolds, and nerve cell proliferation was observed. Their results showed that PCLF-PPy scaffolds are necessary for nerve tissue engineering applications. Progress in studies on polyaniline (PANI) applications in tissue engineering is slower than that of PPy. Fiber web morphology, molecular structure, and electro-active properties have been studied. The results showed the potential of prepared composite for tissue engineering. However, some limitations in the composition of the blended system have occurred due to the necessity of optimization for the PHB: PANI solvent ratio. This helps in obtaining reasonable spinnability of composites. Furthermore, a small amount of PANI leads to alterations in the supermolecular structure of PHB/PANI nanofibers [28].

Each type of scaffold material offers distinct advantages and disadvantages in TE. Natural materials are ideal because of their biocompatibility, low toxicity, and ability to stimulate regeneration in a tissue [29,30]. However, a disadvantage is their inconsistency from batch to batch. Synthetic materials are more consistent and accessible to modify and may lack that intrinsic bioactivity seen in natural scaffolds. Composite scaffolds take the best features of both natural and synthetic scaffolds. However, manufacturing biocomposite scaffolds is complex and expensive [29]. One of the challenges in fabricating tissue engineering scaffolds is determining the appropriate properties for the scaffold, such as high porosity, orientation, high surface area, controlled degradation rate, good mechanical properties, and biocompatibility. The material selection is crucial to determine the most suitable degradation rates for the scaffold, as rapid or delayed degradation may disturb the regeneration process. Further, composition plays a significant part in the properties of nanofibers. The nanofibers may have better hydrophilicity properties and degradation rates by combining materials. In order to produce nanofiber scaffolds with good characteristics, the processing parameters of the characterization tool must be well understood.

On the other hand, the immune response is progressively more recognized as a factor affecting tissue regeneration. Generally, the immune reaction to an implant initiates with a critical response to the wound and native site recognition of foreign materials; subsequently, the chronic immune response involves a particular recognition of antigens or transplanted cells, eventually leading to the rejection of the scaffold or the implant. Each component of the immune response and strategies includes material design, immune cell recruitment/transplantation, anti-inflammatory cytokine delivery, and the local immune response to elevate regeneration. The surface morphology of electrospun scaffolds should also be modified to mimic the natural bone.

Further advances in technology and advanced materials can improve scaffold biocompatibility in vivo. Moreover, bioinspired materials for TE or regenerative medicine require a balance between technical advancement and ethical considerations. A broad perspective exploring toxicity in vivo, environmental impact, and ethical issues within bioinspired materials are much needed to ensure these materials’ biocompatibility and safety concerns, which is a pivotal challenge [31].

## 3. Scaffold Fabrication Techniques

In addition to the material used in the scaffold, the fabrication method is also fundamental in tissue engineering [30]. Scaffold fabrication is the process ‘by which scaffolds with a continuous, uninterrupted pore structure can be made’ [32]. Scaffold architecture is essential for bioactivity, particularly at the micro- and nanoscale. Features like pore size, shape, and interconnectivity directly impact cellular behavior, affecting how cells adhere, migrate, and proliferate on the scaffold. For example, larger interconnected pores improve nutrient and oxygen flow, supporting cell health and metabolic functions. Additionally, micro- and nanoscale structures provide physical signals that can encourage or discourage specific cell differentiation, guiding stem cells toward paths like bone or cartilage formation.

However, a significant challenge in scaffold design still remains due to ineffective cell–scaffold interaction, as cell adhesion, migration, and proliferation vary greatly between synthetic and natural materials. Natural materials often provide better cellular compatibility due to their similarity to the extracellular matrix (ECM), while synthetic materials may lack essential bioactive cues. To overcome these limitations, surface modification techniques, such as coating with ECM proteins or functional biomolecules, are employed to improve cell adhesion and promote cellular integration. These functionalization strategies are especially critical in synthetic scaffolds, enhancing their ability to support stable, organized tissue growth. By closely mimicking the natural extracellular matrix (ECM), these controlled architectural elements foster the development of stable, functional tissues.

**Figure 2:**
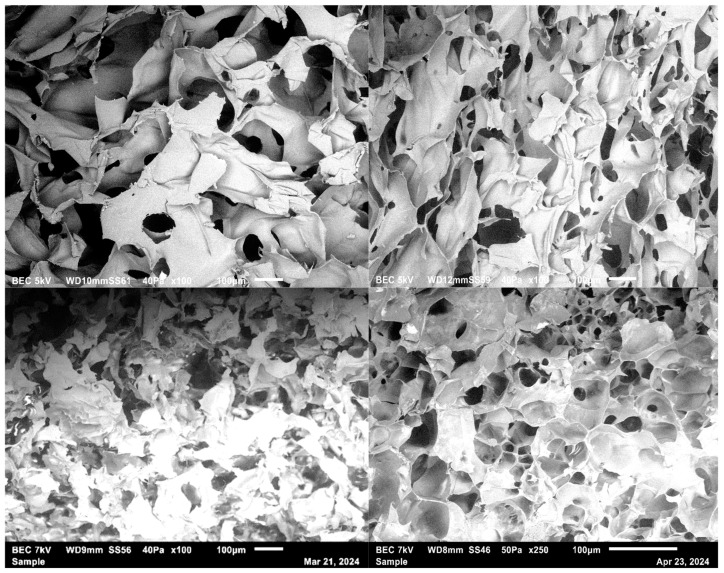
Scanning electron micrograph of pectin/chitosan and Gelatin scaffold fabricated using the technique.

A traditional scaffold fabrication method is solvent casting, where a polymer solution is poured into a mold, and the solvent is evaporated to form a scaffold. This method allows the fabrication of the desired architecture [33]. Another traditional method is gas foaming, a method of fabrication of synthetic scaffolds that avoids solvents. In this method, a polymer is compressed under high temperatures and molded into a solid disc. This disc will be placed in a carbon dioxide chamber for several days. This process allows gas to infiltrate the polymer disc and create a porous structure [34]. Thermally-induced phase separation (TIPS) and freeze drying are other promising techniques for fabricating scaffolds from natural or synthetic polymers [35]. Highly porous and interconnected composite scaffolds were fabricated using a freeze-drying technique. This technique effectively produces composite scaffolds due to the ability to incorporate bioactive materials or drugs (Figure 2).

**Figure 3:**
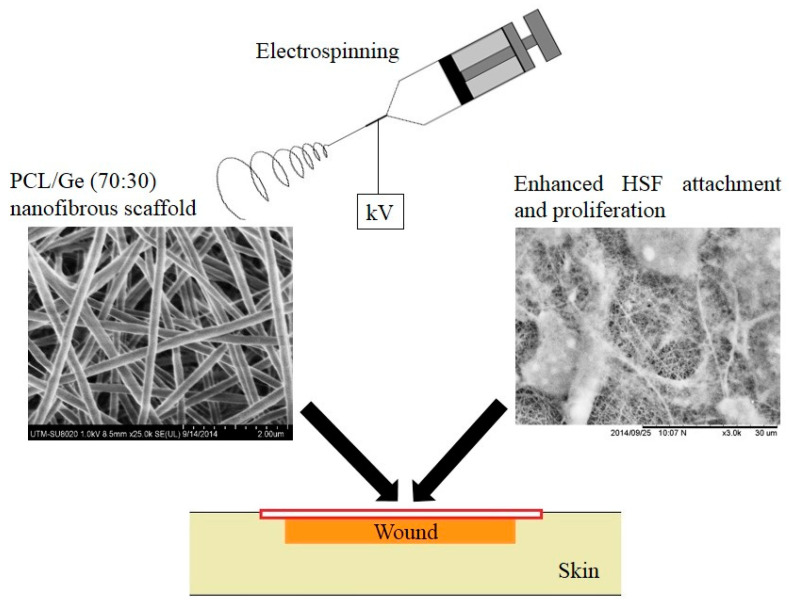
Morphology of electrospun composite scaffold and HSF cell growth after three days of culture on Polycaprolactone/Gelatin scaffold (reproduced from [38]).

3D printing and electrospinning are advanced methods of tissue scaffold fabrication. These techniques are believed to craft intricate scaffolds with enhanced characteristics compared to traditional methods [36]. 3D printing is an additive manufacturing (AM) technique for fabricating structures and complex geometries from three-dimensional (3D) model data. The process consists of printing successive layers of materials formed on each other. Despite allowing for a broader range of geometries, 3D-printed polymer scaffolds tend to have inferior mechanical properties compared to scaffolds fabricated through other methods. Electrospinning is a fabrication technique that uses an electric field to propel and spin a polymer solution into nanofibers on a substrate [35]. Electrospinning is valued as it creates structures with higher surface area-to-volume ratios, which is beneficial in tissue engineering [37]. Combining 3D printing and electrospinning when creating a scaffold can make structures superior to those when the methods are used alone. Figure 3 shows the morphology of the electrospun nanofibrous scaffold and the growth of human skin fibroblast (HSF) cells on the scaffold [38].

Considering the function of the scaffold to direct cellular behaviors such as proliferation, migration, and differentiation, there are a few requirements of the scaffold that must be met. These requirements discussed earlier, such as a porous structure, large pore sizes, and a uniform interconnectedness of the pores, can be met using nanotechnology. Nanotechnology can manipulate a scaffold at the atomic, molecular, and macromolecular levels [38]. In addition, the scaffold can be constructed using the specific geometrical and topological structures found in natural tissue. The use of nanotechnology in scaffolds has been connected to higher mechanical strength, porosity, and enhanced biocompatibility [39].

Among various fabrication techniques, electrospinning has gained broad interest among researchers in producing fibers with controlled diameter, high surface area, and porous interconnected structure. Electrospinning techniques have demonstrated higher efficacy and precision in replicating the geometries seen in tissue. The technique has produced long and continuous nanofiber structures by applying a strong electrostatic field to the polymer solution driven at a high-voltage supply between a needle tip and the collector. The advantages of electrospinning are the production of long, uniform, and continuous nanofibers with a broad range of diameter compared to other techniques. In addition to its simplicity of operation, the technique can produce nanofiber with a controlled diameter and a high surface area-to-volume ratio. This allows better cell incorporation and perfusion that mimic the structure of ECM. The nanofibers also can be produced using multiple polymers and bioactive ingredients.

Although electrospinning was described as a simple technique, there are processing parameters that can physically influence the structure of fiber formed using this technique. Below are the parameters that influence the morphology and diameter of the electrospun fibers [40]:

1. The solution properties such as the type of polymer, the polymer chain conformation, viscosity or concentration, elasticity, polarity, electrical conductivity, and surface tension of the solvent
2. The processing conditions, such as the strength of applied voltage, the distance between the spinneret and the collector, the flow rate for the polymer solution
3. The ambient parameters, such as humidity and temperature of the surroundings

With reference to Table 3, many previous studies of electrospinning have shown how these parameters can significantly influence the formation of fibers. One can obtain the desired structure of smooth nanofibers by adjusting and controlling the appropriate parameters.

Traditional methods, although quicker and more straightforward, lack the complexities required in a scaffold for biomimicry. These methods, such as solvent casting and gas foaming, are still the most cost-effective since advanced methods require expensive equipment. Therefore, even though advanced methods for scaffolding are more capable of mimicking the complex structures found in vivo, the cost problem remains a significant barrier to their widespread adoption in tissue engineering [36]. With the current technologies developed to fabricate tissue-engineered scaffolds, selecting the fabrication technique is very important in designing scaffolds with specific morphological features and cell functions that can provide temporary support for the intended application. Different fabrication techniques have their advantages and disadvantages. Thus, a novel technique is needed to overcome their drawback. The electrospinning fabrication technique can fabricate a nanofiber scaffold with a structure similar to the natural ECM. Although this technique limits the scaffold to only a 2D structure, the nanofiber is complemented with the mimicking feature of the ECM, thus enabling cell growth onto the scaffold. Research is moving towards fabricating 3D microstructure using this technique (Table 4).

While 3D bioprinting has shown extensive application in biomedical and tissue engineering fields, its static and inert nature only considers the earlier state of the printed object. However, in 4D bioprinting, which is integrated with 3D bioprinting, where ‘time’ is integrated as the fourth dimension [49], biocompatible responsive cells or materials can change their functions with time depending on the imposed external stimulus. Due to the development in printing stimulus-responsive materials, which can alter their shape or be able to restructure with cellular self-organization, 4D bioprinting has promising applications in tissue engineering and drug delivery. It was reported that 4D printing allows the fabrication of dynamic constructions that can change property, shape, and functionality over time in response to stimuli, leading to various novel innovations in the tissue engineering field. With intelligent biomaterials, biological components, and living cells, 3D constructs with 4D impacts have been applied to develop smart and dynamic cell-laden TE constructs such as cartilage, bone, and vascular systems. However, 4D bioprinting is a complex system that faces several challenges that can be mitigated by multidisciplinary strategies to address the current issues of basic research in TE and regenerative medicine [50]. Geometrically complex and highly customized structures can be fabricated using 4D printing [51]. Recently, shape memory polymers (SMPs) have received much attention. SMPs are smart polymers that can maintain a temporary shape and return to their original shape when exposed to external stimuli, such as magnetic field, heat, light, and stress. Poly D, L-lactide-co-trimethylene carbonate (PDLLA-co-TMC) is an example of temperature-responsive amorphous SMP. The physically crosslinked SMPs are called thermoplastics; they include polycaprolactone (PCL), polylactic acid (PLA), and thermoplastic polyurethane (PU). Chemically crosslinked SMPs are called thermosetting; they include cyanate resin, epoxy resin, thermosetting PU, polystyrene, and polyimide. Currently, 4D bioprinting technology includes inkjet printing, fused deposition modeling (FDM), direct ink writing (DIW), digital light processing (DLP), stereolithography apparatus (SLA), selective laser sintering (SLS), and more [52]. Table 5 shows the recent research related to 4D bioprinting, which uses smart polymers for biomedical and TE applications.

In general, freeze-drying is a promising bone or cartilage repair fabrication route. RP prototyping, such as 3D Printing or SLS, can also be applied to bone TE scaffolds. Electrospun nanofibers favor regenerating soft tissue such as skin and vascular grafts. Each scaffold fabrication technique has its merits and drawbacks. Since the freeze-drying technique relies on low temperatures and the complete removal of solvent after solidification, the method is suitable for bioactive molecules and drugs, particularly heat-sensitive ones. The method allows the fabrication of interconnective and highly porous scaffolds (>95%), is inexpensive, and is feasible by which the morphological properties of the produced scaffolds can be tuned. This is possible by manipulating process parameters such as polymer type and concentration, cooling rate, solvent/non-solvent ratio, and surfactant presence. A significant drawback of this technique is the usage of organic solvents that should be entirely removed to avoid their harmful outcomes. On the other hand, electrospinning produces nanofibrous scaffolds of high surface area/volume ratio, and combining with the tunable morphological properties of the scaffolds, such as pore size and mechanical strength, exhibits the benefits of the process in TE. However, electrospinning has limited application in polymer type, and residual solvent may affect the biological properties of the scaffolds. Advantages of rapid prototyping techniques such as selective laser sintering (SLS) or 3D bioprinting include the shorter time needed to reach satisfactory prototypes and the reduced trial-and-error stage in scaffold design and construction. However, the toxicity of binder liquids inflicts health restrictions, and poor resolution of the techniques limits their usage in TE constructs. However, 4D bioprinting, by mimicking 3D organs, could solve the problem of the inadequate supply of organ transplants.

## 4. Tissue-Engineered Product Applications

Researchers addressed the limitations of collagen (Col) hydrogels, which tend to have poor physicochemical and mechanical properties, by adding mesoporous bioactive glass nanoparticles. (mBGns) [39]. These Col–mBGn nanocomposite hydrogel scaffolds demonstrated excellent stability due to increased chemical bonding between collagen molecules. Furthermore, mechanical strength was increased, as the Col–mBGn hydrogel scaffolds had improved loading and stiffness compared to those without mBGns [37].

The influential role of mBGns in collagen scaffolds is further demonstrated by the seeding of mesenchymal stem cells. (MSCs) MSCs cultivated within the Col–mBGn hydrogels demonstrated viability and enhanced cytoskeleton extensions in hydrogels with mBGn added. In addition, while Col hydrogen demonstrated extreme shrinkage within a few days of culture, Col–mBGn hydrogels demonstrated no observable shrinkage over 21 days [37].

In one study, Alshammari et al., 2023, explored the relationship of scaffold pore architecture to bone tissue formation using mechanobiological modeling [39]. Two scaffold designs were used: IREG1 and REG. IREG1. Irregular pore architecture had larger pores, measuring 0.4 × 0.2 mm, while REG (regular pore architecture) had smaller pores, measuring 0.3 × 0.2 mm. In the earlier period of the study, the IREG1 scaffold resulted in more considerable bone growth, while in the later period, the REG scaffold resulted in more considerable bone growth than IREG1 [39]. These results suggest that scaffold design and pore architecture can significantly influence bone growth rates over time, highlighting the importance of tailoring scaffold designs for different phases of bone regeneration.

Researchers have used regenerative medicine strategies for decades to develop tissue-engineered vascular grafts (TEVG) [60]. The TEVG was developed by seeding tubular biodegradable polymeric scaffolds with autologous bone marrow-derived mononuclear cells. In the first-in-human study, the feasibility of TEVGs was demonstrated as the TEVG transformed into a living vascular graft that could grow. However, this initial clinical trial revealed a high incidence of early TEVG stenosis, preventing this promising technology’s widespread use [60]. Researchers have used mouse and computational models to address this complication and design strategies to inhibit stenosis. Recently, these strategies have been validated in large animal models and clinics via an FDA-approved clinical trial [60]. This case highlights the challenges faced in using tissue-engineered vascular grafts. It demonstrates the effective solutions developed, underscoring this innovative technology’s continued promise and potential.

The scaffold’s pore size or diameter is an important parameter controllable by the existing techniques. The MSCs growth was reported to be significantly higher when the average pore diameter was 300 µm, and chondrogenic markers were upregulated in scaffolds with 300 µm with a porosity range of 73% to 86%. On the other hand, human skin fibroblasts exhibited higher viability in scaffolds with pore diameters of 74–160 µm. Osteoblast cells for bone regeneration showed better ALP activity in the scaffold with larger pore sizes from 500 µm to 1100 µm with 70% porosity. The 370 to 400 µm pore diameter range was the most effective for the chondrogenic differentiation from the adipose stem cells [61]. As the transport properties in the scaffolds with smaller pore sizes are lowered due to the lack of flux of the bloodstream or the interstitial fluid that delivers nutrients to cells, an optimal range of pore diameter would ensure appropriate outcomes.

As the bulk material property, an increase in the scaffold’s porosity is associated with a decrease in Young’s modulus. Most scaffolds using the commonly existing technique have porosities between 70% to 90%. Scaffolds with lower porosity have a larger surface area that shows better initial cell attachment. In contrast, scaffolds with a higher porosity are linked to increased diffusivity of nutrients and higher permeability, but the low Young’s modulus reduces the mechanical properties of the scaffold. The scaffolds fabricated from biodegradable materials that lose their mechanical integrity during degradation can be partly counterbalanced by the newly synthesized ECM formation inside the scaffolds, which will further reinforce the overall mechanical properties of the scaffold [61].

The degradation mechanism and rate of biodegradable polymers can be affected by numerous factors [62]. Among the factors that affect degradation are molecular weight, structure and content of the comonomer unit, crystallinity, orientation, blending, porosity, pH, temperature, and catalytic molecules or ions. The uses of several polymers could be extended considerably if a broader range of degradation profiles was available. Bone growth and healing can be stimulated theoretically as the polymer slowly degrades, avoiding replacement operations. Natural PHBV copolymers are hydrolyzed in water with the normal universal acid-base catalysis for esters. The degradation rate is relatively rapid at high pH, but hydrolysis proceeds very slowly in a neutral buffer at body temperature.

Moreover, the experiments suggested that the rate of degradation of PHBV in vivo is significantly faster than the in vitro hydrolysis rate at the same temperature and pH. The nonspecific esterase and lysozyme enzymes secreted by the body’s immune system catalyze the process. The variation of the degradation rate of PHBV in vivo with the activity of the body’s immune system is thus helpful in explaining some of the discrepancies in the literature on PHBV biodegradation. The range of biodeterioration of implanted films can vary from very rapid to modest but measurable resorption to virtually undetectable weight loss of fiber monofilament over 18 months. It was also reported that PHBV and cell/PHBV constructs can produce neocartilage in a heterotopic site, although the degradation rates of PHBV in different environments need more investigation [62]. In general, the hydrolytic degradation of semicrystalline high molecular weight PLLA proceeds through random bulk hydrolysis in two distinct stages. The first stage is characterized by the preferential attack of the ester linkages in the more accessible amorphous regions, while the second stage is characterized by the attack of the less accessible crystalline regions. It was reported that the cleavage of an ester bond of PLGA polymers yielded a carboxyl end group and a hydroxyl one, and thus formed carboxyl end groups could catalyze the hydrolysis of other ester bonds. This phenomenon is called autocatalysis. The autocatalysis rate equation is applicable when the extent of the reaction is slow or before the specimen experiences significant weight loss [62].

Using quantitative analysis, the biocompatibility of freeze-cast tissue scaffolds was tested for several materials in mice. The freeze-cast method is versatile in creating porous and structured scaffolds. In the quantitative analysis, measurements were taken for encapsulation thickness, leukocyte cell counts, and density (of the scaffolds). Furthermore, researchers did lymphocyte assays, counting the number of capillaries and histological scoring. All of these methods were used to grade the biocompatibility of the scaffolds based on the cell–matrix and cell–cell interactions [63]. All of these scaffolds were derived from natural sources, and some had chemical modifications on top of them. In vivo biocompatibility was tested using mice, and all showed to be biocompatible. However, some differed in histopathological responses. This study outlines future directions and suggests a focus on systematically investigating the effects of composition and processing parameters on the response to the scaffold [63].

## 5. Importance of Continued Innovation in Scaffold Development Using Smart Materials

The case studies reviewed underscore the ongoing need for innovation in scaffold development [60]. The TEVG example demonstrates that overcoming clinical challenges such as stenosis requires a multidisciplinary approach, integrating insights from small and large animal models, computational simulations, and clinical trials [60]. The continued promise of tissue-engineered scaffolds in regenerative medicine highlights the importance of sustained research and development efforts. By addressing current limitations and exploring new materials and techniques, the field can advance toward creating effective, biocompatible scaffolds for various tissue engineering applications.

The field of TE has made substantial progress in developing scaffolds that support tissue regeneration. Advances in materials science and fabrication technologies have enabled the creation of scaffolds that closely mimic the complex structures of natural tissues. Despite these advancements, challenges remain, particularly in achieving consistent biocompatibility and mechanical properties while managing cost and scalability. A suitable TE product should be non-toxic to cells, maintain cell proliferation and differentiation, control antimicrobial activities, and possess electrical stimulation properties both in vitro and in vivo [64]. Owing to the excellent biological and physicochemical characteristics, functionalized MXene exhibits biocompatibility, low toxicity, antibacterial activity, and electrical stimuli, as well as high surface area and hydrophilicity [65]. MXene-incorporated nanocomposites of the various nanostructured membranes, scaffolds, nanofiber, polymer-based microspheres, and 3D-printed devices can be fabricated using different techniques [64]. There is an enormous and unexplored possibility of functionalized MXene nanosheets and MXene-incorporated nanocomposites for TE applications. Biocompatibility, elevated targeting capacity, selectivity, stability, and controlled release behaviors of MXene-based constructs are needed for TE applications.

## 6. Tissue Engineering and the Use of Nanomaterials

As TE is a field within bioengineering that applies cells, materials, and engineering methods to create or repair tissues and organs, accomplishing the primary goal of creating and repairing tissues is crucial in addressing issues such as organ shortages, tissue damage, and disease. A key obstacle in tissue engineering is the creation of tissues that can mimic the function and microenvironments of native tissues. This challenge stems from complex tissue structures having specific biochemical, physical, and mechanical properties and are difficult to replicate in tissues grown in a lab. The innovative use of nanomaterials in tissue engineering has the potential to address this limitation. Nanomaterials are chemical substances or materials with nanoscale dimensions. These materials are favorable because they can interact with biological systems in a controlled manner and on a small scale. With nanomaterials, scientists can design scaffolds that can support cells and guide cellular behavior in ways that resemble a natural tissue environment. While nanomaterials have been used in tissue engineering for over two decades, their full potential in this field remains unknown [66].

Further research is needed to understand and optimize the use of nanomaterials in different tissue types, whether soft tissue-like skin or rigid skin. The full potential of nanomaterials can be used to mimic the structure of native tissues by fine-tuning the properties of these nanomaterials, such as shape, structure, and size. Nanomaterials can mimic tissue structures because biological tissues comprise nanoscale components such as proteins, lipids, and other molecules that comprise the extracellular matrix (ECM).

The ECM provides structural support for cells and has a critical role in cell signaling. We have proper cell growth, migration, and differentiation with the ECM. Therefore, we must create scaffolds that can truly replicate the ECM, as it is vital to the success of engineered tissues. This property significantly bridges the gap between tissues created in vivo and in vitro. Furthermore, nanomaterials such as nanoparticles demonstrate toughness and a high surface area to volume. These two properties are essential for tissue engineering because they allow nanomaterials to withstand the mechanical stress and exchange of substances that engineered tissues experience in vivo. These properties are also important in facilitating cell–matrix interactions and cell proliferation. For example, gold nanoparticles (GNPs) and titanium dioxide nanoparticles have enhanced cell proliferation rates in bone and cardiac tissue [67].

Nanoparticles are only one of a variety of nanomaterials used in tissue engineering. Each nanomaterial type has unique properties that make it suitable for different applications. By understanding the strengths of each material, researchers can choose the most appropriate one for the specific tissue they are engineering. Other nanomaterials, such as nanofibers, nanocomposites, and nanotubes, have also proven useful. Nanoparticles are a specific type of nanomaterial, typically spherical, and they possess at least one dimension within the nanoscale range. They are often used in drug delivery, imaging, and diagnostic applications. Their small size allows for precise delivery of drugs or biomolecules to specific areas within the body, which is particularly useful in targeted therapies for tissue regeneration. Due to their small size and high surface area, nanoparticles are ideal for tissue engineering applications [68,69]. This high surface area enhances their interaction with cells, making nanomaterials more effective at promoting cell attachment and growth than larger-scale materials. Nanofibers mimic the architecture of natural tissues and enhance the scope of scaffold fabrication. The ability to closely resemble the fibrous structure of the extracellular matrix makes nanofibers particularly useful in creating scaffolds that support the growth of various cell types. Their structure allows for a highly porous network, facilitating the exchange of nutrients and waste products between the cells and their environment. These fibers exhibit a high surface area to volume ratio, which is essential for the absorptive function of tissues.

Additionally, nanofibers have a microporous structure that benefits tissue engineering [70]. The porosity of these fibers allows for better cell infiltration, which is necessary to integrate engineered tissues with the surrounding native tissues once implanted.

Nanocomposites are composed of a combination of different nanomaterials, allowing them to have a broader range of properties than pure nanomaterials. By combining different materials, nanocomposites can achieve a balance of properties such as mechanical strength, flexibility, and bioactivity, making them more versatile for use in various tissue engineering applications. For instance, nanocomposites can be designed to simultaneously provide MR imaging contrast and allow for drug delivery, combining the best properties of both materials [71]. This multifunctionality is valuable in applications where the progress of tissue regeneration needs to be monitored in real-time, as it allows for both therapeutic and diagnostic functions in a single scaffold. Carbon nanotubes (CNTs) are especially useful in tissue engineering scaffolds. They can modify tissues’ mechanical strength, conductivity, degradation, and biocompatibility. The ability to fine-tune these properties makes CNTs adaptable to various tissue types, from soft tissues like nerve cells to hard tissues like bone. Furthermore, CNTs can be adjusted to meet the specific requirements of different tissues, making them highly versatile in tissue engineering applications [72]. Their flexibility in design means that CNTs can be engineered to provide the exact mechanical and electrical properties needed for different types of tissues.

## 7. Future Research Directions

Future research should refine scaffold materials to enhance biocompatibility and mechanical properties while reducing immune responses [73,74]. Continued exploration of advanced fabrication techniques, such as integrating 3D printing, 4D printing, and electrospinning, will be crucial for creating more intricate and functional scaffolds [40,49,75,76,77,78,79]. Additionally, understanding the biological mechanisms underlying scaffold–tissue interactions will be essential for optimizing scaffold designs. Research should also aim to develop scalable and cost-effective production methods to facilitate widespread clinical application.

Conductive polymers with remarkable biocompatibility have been researched and used in various biomedical applications [79]. Due to its properties of high electrical conductivity and chemical stability, poly(3,4-ethylenedioxythiophene) (PEDOT) is currently being used in biomedicine and biotechnology. The copolymer of PEDOT with poly(4-styrene sulfonate) (PSS) has good stability, and the PEDOT: PSS is hydrophilic and holds conductivity properties. The electrochemical, thermal, and oxidative stability of PEDOT: PSS is to be used in wide applications in nanocomposites, flexible electrodes, electrochromic displays, and transistors. Positive results showed that conductive polymer scaffolds from PEDOT: PSS are structurally suitable for bone tissue engineering.

On the other hand, the conductive nature of the conducting polymer will allow the cells or tissues cultured upon them to be stimulated [26,79]. Composites are developed by blending conductive polymers with other polymers or materials. Modifying conductive polymers can render these polymers biodegradable and biocompatible, making them very useful in TE applications. Applications of conductive polymers are significant concerns. Due to the toxicity and biocompatibility, extensive studies are still required to apply conductive polymers in tissue regeneration and biomedical devices. Cell-conductive polymer-biomolecule-based scaffold biointerfaces should address the insight of cell interaction with conductive materials. The appropriate knowledge will thus help researchers aim for innovative biomaterials for tissue engineering applications.

Functionalization techniques, including integrating growth factors, genetic materials, or antimicrobial agents, are crucial for enhancing scaffold bioactivity and achieving specific tissue engineering goals. Recent innovations, like sustained-release mechanisms for growth factors, show promise in promoting prolonged cellular responses within scaffold matrices [80]. However, challenges include controlling release rates and preserving factor stability. Incorporating genetic materials into scaffolds has enabled localized gene therapy applications, though complex delivery systems often limit efficiency. Antimicrobial functionalization, such as embedding silver nanoparticles, is also being explored to reduce infection risks in implantable scaffolds. However, this can impact biocompatibility [81]. As these techniques evolve, functionalization strategies are expected to increase scaffold functionality. Further optimization is required to ensure efficacy, compatibility, and longevity in biological environments.

Achieving vascularization within scaffolds is another critical challenge that remains unsolved in tissue engineering. Effective vascular networks are essential for the survival and functionality of large tissue constructs by enabling efficient nutrient and oxygen transport. Innovative strategies, such as embedding angiogenic growth factors, integrating endothelial cells, and developing pre-vascularized scaffold models, have shown potential but require further optimization to replicate the complexity of native vascular systems [82]. Additionally, addressing scalability is paramount as the demand for scaffold-based regenerative therapies grows. Future efforts should focus on developing automated, scalable manufacturing processes that maintain high precision and uniformity across large batches, ensuring that engineered tissues remain viable for clinical applications. Together, these efforts will push the field toward more reliable and widely applicable tissue-engineered solutions.

In order to harness the benefits of biocomposite scaffolds in clinical settings, a thorough understanding of regulatory hurdles should be addressed. Several regulatory considerations should be addressed, including biocompatibility and safety assessment, interactions between cells and nanoparticles, and cell–scaffold interaction. International standards and guidelines to attain a harmonious regulatory approach with the innovations highlighted in molecular safety assessments during development are required [83]. However, standardized assessment methods, balanced innovation, safety, and unique technologies are still challenging.

## 8. Conclusions

This review highlights several significant contributions of biomaterials-based scaffolds to TE and regenerative medicine by providing an overview of existing literature, key findings, and a review of the key features and critical importance of the compatibility of biopolymer scaffolds in providing regenerative improvement to damaged organs. This review identifies the essential findings, highlights them, and contextualizes them within the broader aspect of the research. Innovations in scaffold materials, including natural, synthetic, and composite options, offer distinct advantages and challenges. In TE applications, the nanomaterials not only support the physical structure of the tissue but also interact with cells to promote faster and more efficient tissue regeneration. The choice of fabrication techniques, from traditional methods like solvent casting and gas foaming to advanced techniques such as 3D printing and electrospinning, significantly impacts scaffold efficiency, cost, and suitability for various tissues. From the diverse array of biomaterials utilized in scaffold fabrication to exploring their essential qualities, such as structural integrity and biological properties, this review provides an understanding of the vital role of bio and nanomaterials in fabricating TE scaffolds.

## Figures and Tables

**Table 1:** Applications of some natural polymers in tissue engineering.

Natural Polymer	Structure/Method of Production	Biological Properties	References
Collagen	Fibrillar structure, which contributes to the extracellular scaffolding	Promotes regeneration and angiogenesis of the bone through monocyte immunomodulation	[16]
Chitosan	It contains an amine group, vital in pH sensitivity and functionality	Induces biological activity by showing excellent antimicrobial activity against bacteria	[17]
Gelatin	Composed of a freeze-dried fiber scaffold	Produces a scaffold that is enzymatically crosslinked to enhance bone regeneration.	[18]
Collagen	Consists of a triple helix chain formed by α chains	Offers low immunogenicity, a porous structure, permeability, good biocompatibility, and biodegradability	[16]
Collagen	Classical fibril-forming collagens, including types I, II, and III collagens	Crosslink formation can shield or modify major antigenic sites and, thus, reduce their capacity to interact with antibodies.	[19]
Chitosan	Semicrystalline biopolymer contains several hydrogen bonds forming functional groups, including amino and hydroxyl groups.	The hydrogels are pH-sensitive in aqueous media, so these stimuli-responsive hydrogels are the best choice for drug delivery.	[20]
Chitosan	Scaffolds, cells, and bio-signals together minimize artificial and cellular environment	Cardiovascular tissue engineering	[15]
Gelatin	In its structure, amino acid sequences such as the arginine-glycine-aspartic acid (RGD) motif improve cell adhesion, differentiation, and proliferation.	Obtain different isometric points.	[18]

**Table 2:** A summary of several pectin systems in tissue engineering approach [19].

Pectin Systems	Method	Application
Low-methoxyl citrus pectin	UV photocrosslinking with peptide crosslinkers (cell-degradable) and adhesive ligands (integrin-specific); lyophilization	Skin tissue engineering
Sugar beet pectin (SBP) crosslinked by visible light	Applying 405 nm visible light in the presence of tris(bipyridine)ruthenium (II) chloride hexahydrate and sodium persulfate, rapid hydrogenation of SBP was obtained; 3D hydrogel constructs were obtained using 3D bioprinting	Promising for liver and other soft tissue engineering
Citrus peel’s pectin crosslinked with (3glycidyloxypropyl) trimethoxysilane (GPTMS)	Freeze-drying or 3D bioprinting	Various tissue regeneration
Pectin/chitin/nano CaCO_3_	Lyophilization	Bone regeneration
Pectin/chitosan	Freeze-drying	Bone tissue engineering
Pectin/strontium/hydroxyapatite	Solution-based chemical technique	Bone regeneration
Collagen/polyurethane/pectin	Semi-interpenetration process	Bone regeneration
Pectin/PVA	Freezing–thawing	Bone regeneration
Poly(L-lactide-co-ɛ-caprolactone) (PLCA)/pectin	Scaffolds functionalized with pectin	In vitro and in vivo bone regeneration

**Table 3:** Previous studies based on electrospinning processing parameter.

Parameter	Summary	Reference
Polymer concentration	Adequate chain entanglements from suitable concentrations of the polymer solution can electrospun continuous uniform nanofibers in a strong enough electric field. Fiber diameters increase when the initial polymer concentration increases as jet elongation is slower.	[41]
Solvent volatility	Wet fibers may combine to form a membrane. Slow subsequent evaporation of solvent and tube collapse produced nanofibers with flat ribbon-like shapes derived from the fluid-filled, incompletely dry nanofiber.	[42]
Solution conductivity	When conductivity is increased, many charges can be carried by the electrospinning jet. The addition of ions can increase the conductivity of the solution. With the increased charges solution, the stretching of the solution will increase and will tend to produce a smaller diameter of fibers.	[43]
Applied voltage	When the applied voltage increases, the solution shaped at the needle tip can gradually change into a Taylor cone	[44]
Flow rate	When the flow rate increases, there is a corresponding increase in the fiber diameter or bead size, which is apparent as a greater volume of solution is drawn away from the needle tip. Decreasing flow rate tends to decrease fiber diameter as less fluid is ejected.	[45]
Tip-collector distance	The fiber diameter also decreased when the distances from the Taylor cone increased because the jet elongation time also increased.	[46]

**Table 4:** Strategies in fabricating 3D nanofibers.

Methods	Summary	Reference
Increasing spinning time	In this way, an electrospun fiber membrane with a certain thickness will be obtained, which can reach hundreds of microns and become a 3D fibrous structure, although these methods may take a long time (for example, from 20 min to 20 h) till it grows to a sufficient 3D structure); mat thickness increases by increased spinning times leading to 3D fibrous thickness, and multilayered with different materials can be fabricated by sequential electrospinning and co-electrospinning. This method’s advantages include controlling each layer’s fiber diameter, composition, and porosity.	[47]
Assembly by post-processing of 2D electrospun fibrous structures	Examples are folding, layer-by-layer electrospinning, sintering, mechanical expansion such as peeling off the thin film from the collector, and then bending/folding or stacking the fiber layers into a 3D fibrous structure like pipe or thick mat	[48]
Direct assembly by an auxiliary factor	Examples are a 3D template, liquid, and collector. In addition, 3D fiber structures can also be successfully obtained through modification of the collector, for example, substituting the conventional 2D flat collector with a 3D collecting template and using liquid collection and removing microparticles filled between nanofibers have been reported, although a subsequent treatment to dry the as-prepared 3D structures or handle with the porogen is usually needed.	[40]
Self-assembly	A rapid growth of 3D fibrous macro without any additional assistance. Examples are fibrous yarns or spongiform fiber stacks.	[40]

**Table 5:** 4D Bioprinting for TE applications.

SMPs	Technique/Mechanisms	Applications	Reference
Poly (N-isopropyl acrylamide) hydrogel matrix with 0.8 wt% nano fibrillated cellulose (NFC)	Biomimetic 4D printing. Reversible shape changes in water of varying temperature	Composite hydrogel architectures were 4D printed with localized, anisotropic swelling behavior that solves the inverse problem of designing the alignment patterns for prearranged target shapes generating complex three-dimensional morphologies for generating architectures for biomedical devices, tissue engineering, and soft robotics.	[53]
Bistrips/patches based on a poly(*N*-isopropyl acrylamide)-based hydrogel	Temperature-responsive swelling	A potential route for the development of self-folding stimuli-responsive micro-devices for biomedical applications.	[54]
Polylactic acid (PLA) and continuous carbon fiber-based continuous fiber fiber-reinforced thermoplastic Composites (CFRTPCs)	Fused deposition modeling (FDM)	Light structures in the field of aviation and aerospace and biomedical applications.	[55]
Polybutylene succinate and polylactic acid (PBS/PLA) filament	4D printed and the graphene oxide (GO) functionalized shape memory PBS/PLA scaffolds	4D printed PBS/PLA filament showed outstanding shape memory performance and demonstrated a promising prospect in the biomedical field.	[56]
Semicrystalline thermoplastic PLA pellets and Fe_3_O_4_ nanoparticles	Direct ink writing (DIW)	Minimally invasive medicine, biomedical devices	[57]
Polyethyleneimine (PEI); Hyaluronic acid; Gelatin; Human umbilical vein endothelial cells (HUVECs)	4D inkjet printing	Tissue engineering	[58]
Polyethylene glycol diacrylate (PEGDA) hydrogel	Digital light processing (DLP)	Cardiac tissue regeneration	[59]
Thermoplastic polyurethane	Selective laser sintering (SLS). The shape-recovered scaffold facilitated directional cell adhesion and stimulated cell proliferation.	Bone tissue engineering	[59]

## Data Availability

No new data were created or analyzed in this study. Data sharing is not applicable to this article.

## References

[1] Garot C., Bettega G., Picart C. (2020). Additive Manufacturing of Material Scaffolds for Bone Regeneration: Toward Application in the Clinics. Adv. Funct. Mater..

[2] Aldana A.A., Abraham G.A. (2017). Current advances in electrospun gelatin-based scaffolds for tissue engineering applications. Int. J. Pharm..

[3] Gregor A., Filová E., Novák M., Kronek J., Chlup H., Buzgo M., Blahnová V., Lukášová V., Bartoš M., Nečas A. (2017). Designing of PLA scaffolds for bone tissue replacement fabricated by ordinary commercial 3D printer. J. Biol. Eng..

[4] Mao A.S., Mooney D.J. (2015). Regenerative medicine: Current therapies and future directions. Proc. Natl. Acad. Sci. USA.

[5] Dzobo K., Thomford N.E., Senthebane D.A., Shipanga H., Rowe A., Dandara C., Pillay M., Motaung K.S.C.M. (2018). Advances in Regenerative Medicine and Tissue Engineering: Innovation and Transformation of Medicine. Stem Cells Int..

[6] Kaul H., Ventikos Y. (2015). On the genealogy of tissue engineering and regenerative medicine. Tissue Eng. Part B Rev..

[7] Krishani M., Shin W.Y., Suhaimi H., Sambudi N.S. (2023). Development of Scaffolds from Bio-Based Natural Materials for Tissue Regeneration Applications: A Review. Gels.

[8] Marques C.F., Diogo G.S., Pina S., Oliveira J.M., Silva T.H., Reis R.L. (2019). Collagen-based bioinks for complex tissue engineering applications: A comprehensive review. J. Mater. Sci. Mater. Med..

[9] Silver F.H., Jaffe M., Shah R.G. (2018). Structure and behavior of collagen fibers. Handbook of Properties of Textile and Technical Fibers.

[10] Fan J., Abedi-Dorcheh K., Sadat Vaziri A., Kazemi-Aghdam F., Rafieyan S., Sohrabinejad M., Ghorbani M., Rastegar Adib F., Ghasemi Z., Klavins K. (2022). A Review of Recent Advances in Natural Polymer-Based Scaffolds for Musculoskeletal Tissue Engineering. Polymers.

[11] Afewerki S., Sheikhi A., Kannan S., Ahadian S., Khademhosseini A. (2018). Gelatin-polysaccharide composite scaffolds for 3D cell culture and tissue engineering: Towards natural therapeutics. Bioeng. Transl. Med..

[12] Kuttappan S., Mathew D., Nair M.B. (2016). Biomimetic composite scaffolds containing bioceramics and collagen/gelatin for bone tissue engineering—A mini-review. Int. J. Biol. Macromol..

[13] Lavanya K., Chandran S.V., Balagangadharan K., Selvamurugan N. (2020). Temperature- and pH-responsive chitosan-based injectable hydrogels for bone tissue engineering. Mater. Sci. Eng. C Mater. Biol. Appl..

[14] Islam S., Bhuiyan M.A.R., Islam M.N. (2017). Chitin and Chitosan: Structure, Properties and Applications in Biomedical Engineering. J. Polym. Environ..

[15] Ahmed S., Annu Ali A., Sheikh J. (2018). A review on chitosan-centred scaffolds and their applications in tissue engineering. Int. J. Biol. Macromol..

[16] Cen L., Liu W.E.I., Cui L.E.I., Zhang W., Cao Y. (2008). Collagen Tissue Engineering: Development of Novel Biomaterials and Applications. Pediatr. Res..

[17] Gholap A.D., Rojekar S., Kapare H.S., Vishwakarma N., Raikwar S., Garkal A., Mehta T.A., Jadhav H., Prajapati M.K., Annapure U. (2024). Chitosan scaffolds: Expanding horizons in biomedical applications. Carbohydr. Polym..

[18] Echave M.C., Pimenta-Lopes C., Pedraz J.L., Mehrali M., Dolatshahi-Pirouz A., Ventura F., Orive G. (2019). Enzymatic crosslinked gelatin 3D scaffolds for bone tissue engineering. Int. J. Pharm..

[19] Sultana N. (2023). Biological Properties and Biomedical Applications of Pectin and Pectin-Based Composites: A Review. Molecules.

[20] Dong C., Lv Y. (2016). Application of Collagen Scaffold in Tissue Engineering: Recent Advances and New Perspectives. Polymers.

[21] Wong S.K., Yee MM F., Chin K.-Y., Ima-Nirwana S. (2023). A review of the application of natural and synthetic scaffolds in bone regeneration. J. Funct. Biomater..

[22] Sowmya S., Bumgardener J.D., Chennazhi K.P., Nair S.V., Jayakumar R. (2013). Role of nanostructured biopolymers and bioceramics in enamel, dentin and periodontal tissue regeneration. Prog. Polym. Sci..

[23] Prabhakaran M.P., Venugopal J., Chan C.K., Ramakrishna S. (2008). Surface modified electrospun nanofibrous scaffolds for nerve tissue engineering. Nanotechnology.

[24] Turnbull G., Clarke J., Picard F., Riches P., Jia L., Han F., Li B., Shu W. (2018). 3D bioactive composite scaffolds for bone tissue engineering. Bioact. Mater..

[25] Lari A., Sun T., Sultana N. (2016). PEDOT:PSS-Containing Nanohydroxyapatite/Chitosan Conductive Bionanocomposite Scaffold: Fabrication and Evaluation. J. Nanomater..

[26] Chang H.C., Sun T., Sultana N., Lim M.M., Khan T.H., Ismail A.F. (2016). Conductive PEDOT:PSS coated polylactide (PLA) and poly(3-hydroxybutyrate-co-3-hydroxyvalerate) (PHBV) electrospun membranes: Fabrication and characterization. Mater. Sci. Eng. C.

[27] Moroder P., Runge M.B., Wang H., Ruesink T., Lu L., Spinner R.J., Windebank A.J., Yaszemski M.J. (2011). Material properties and electrical stimulation regimens of polycaprolactone fumarate–polypyrrole scaffolds as potential conductive nerve conduits. Acta Biomater..

[28] Mattioli-Belmonte M., Giavaresi G., Biagini G., Virgili L., Giacomini M., Fini M., Giantomassi F., Natali D., Torricelli P., Giardino R. (2003). Tailoring biomaterial compatibility: In vivo tissue response versus in vitro cell behavior. Int. J. Artif. Organs.

[29] Gang F., Ye W., Ma C., Wang W., Xiao Y., Liu C., Sun X. (2023). 3D printing of PLLA/biomineral composite bone tissue engineering scaffolds. Materials.

[30] Kaviani M., Geramizadeh B. (2023). Basic Aspects of Skin Tissue Engineering: Cells, Biomaterials, Scaffold Fabrication Techniques, and Signaling Factors. J. Med. Biol. Eng..

[31] Singh A.V., Chandrasekar V., Prabhu V.M., Bhadra J., Laux P., Bhardwaj P., Al-Ansari A.A., Aboumarzouk O.M., Luch A., Dakua S.P. (2024). Sustainable bioinspired materials for regenerative medicine: Balancing toxicology, environmental impact, and ethical considerations. Biomed. Mater..

[32] Kundu J., Pati F., Shim J., Cho D. (2014). Rapid prototyping technology for bone regeneration. Rapid Prototyping of Biomaterials.

[33] Joseph B., Jose C., Kavil S.V., Kalarikkal N., Thomas S. (2023). Functional Biomaterials: Design and Development for Biotechnology, Pharmacology, and Biomedicine.

[34] Capuana E., Lopresti F., Carfì Pavia F., Brucato V., La Carrubba V. (2021). Solution-Based Processing for Scaffold Fabrication in Tissue Engineering Applications: A Brief Review. Polymers.

[35] Sultana N., Wang M. (2008). Fabrication of HA/PHBV composite scaffolds through the emulsion freezing/freeze-drying process and characterization of the scaffolds. J. Mater. Sci. Mater. Med..

[36] Ejiohuo O. (2023). A Perspective on the Synergistic Use of 3D Printing and Electrospinning to Improve Nanomaterials for Biomedical Applications. Nano Trends.

[37] El-Fiqi A., Lee J.H., Lee E.J., Kim H.W. (2023). Collagen hydrogels incorporated with surface-aminated mesoporous nano bioactive glass: Improved physicochemical stability and mechanical properties are adequate for complex tissue engineering. Acta Biomater..

[38] Lim M.M., Sun T., Sultana N. (2015). In Vitro Biological Evaluation of Electrospun Polycaprolactone/Gelatine Nanofibrous Scaffold for Tissue Engineering. J. Nanomater..

[39] Alshammari A., Alabdah F., Wang W., Cooper G. (2023). Virtual Design of 3D-Printed Bone Tissue Engineered Scaffold Shape Using Mechanobiological Modeling: Relationship of Scaffold Pore Architecture to Bone Tissue Formation. Polymers.

[40] Sun B., Long Y., Zhang H., Li M., Duvail J., Jiang X., Yin H. (2014). Advances in three-dimensional nanofibrous macrostructures via electrospinning. Prog. Polym. Sci..

[41] Hong Y. (2016). Electrospun fibrous polyurethane scaffolds in tissue engineering. Advances in Polyurethane Biomaterials.

[42] Koombhongse S., Liu W., Reneker D.H. (2001). Flat polymer ribbons and other shapes by electrospinning. J. Polym. Sci. Part B Polym. Phys..

[43] Abdulhussain R., Adebisi A., Conway B.R., Asare-Addo K. (2023). Electrospun nanofibers: Exploring process parameters, polymer selection, and recent applications in pharmaceuticals and drug delivery. J. Drug Deliv. Sci. Technol..

[44] Deitzel J., Kleinmeyer J., Harris D., Beck Tan N. (2001). The effect of processing variables on the morphology of electrospun nanofibers and textiles. Polymer.

[45] Zong X., Kim K., Fang D., Ran S., Hsiao B.S., Chu B. (2002). Structure and process relationship of electrospun bioabsorbable nanofiber membranes. Polymer.

[46] Li Y., Zhu J., Cheng H., Li G., Cho H., Jiang M., Gao Q., Zhang X. (2021). Developments of Advanced Electrospinning Techniques: A Critical Review. Adv. Mater. Technol..

[47] Soliman S., Pagliari S., Rinaldi A., Forte G., Fiaccavento R., Pagliari F., Traversa E. (2010). Multiscale three-dimensional scaffolds for soft tissue engineering via multimodal electrospinning. Acta Biomater..

[48] Subramanian A., Krishnan U.M., Sethuraman S. (2011). Fabrication of uniaxially aligned 3D electrospun scaffolds for neural regeneration. Biomed. Mater..

[49] Gao B., Yang Q., Zhao X., Jin G., Ma Y., Xu F. (2016). 4D Bioprinting for Biomedical Applications. Trends Biotechnol..

[50] Lai J., Liu Y., Lu G., Yung P., Wang X., Tuan R.S., Li Z.A. (2024). 4D bioprinting of programmed dynamic tissues. Bioact. Mater..

[51] Yan S., Zhang F., Luo L., Wang L., Liu Y., Leng J. (2023). Shape Memory Polymer Composites: 4D Printing, Smart Structures, and Applications. Research.

[52] Gladman A.S., Matsumoto E.A., Nuzzo R.G., Mahadevan L., Lewis J.A. (2016). Biomimetic 4D printing. Nat. Mater..

[53] Ionov L. (2014). Hydrogel-based actuators: Possibilities and limitations. Mater. Today.

[54] Tian X., Liu T., Yang C., Wang Q., Li D. (2016). Interface and performance of 3D printed continuous carbon fiber reinforced PLA composites. Compos. Part A Appl. Sci. Manuf..

[55] Lin C., Liu L., Liu Y., Leng J. (2022). 4D printing of shape memory polybutylene succinate/polylactic acid (PBS/PLA) and its potential applications. Compos. Struct..

[56] Wei H., Zhang Q., Yao Y., Liu L., Liu Y., Leng J. (2017). Direct-write fabrication of 4D active shape-changing structures based on a shape memory polymer and its nanocomposite. ACS Appl. Mater. Interfaces.

[57] Cui C., Kim D.O., Pack M.Y., Han B., Han L., Sun Y., Han L.H. (2020). 4D printing of self-folding and cell-encapsulating 3D microstructures as scaffolds for tissue-engineering applications. Biofabrication.

[58] Wang Y., Cui H., Wang Y., Xu C., Esworthy T.J., Hann S.Y., Boehm M., Shen Y.L., Mei D., Zhang L.G. (2021). 4D printed cardiac construct with aligned myofibers and adjustable curvature for myocardial regeneration. ACS Appl. Mater. Interfaces.

[59] Shuai C., Wang Z., Peng S., Shuai Y., Chen Y., Zeng D., Feng P. (2022). Water-responsive shape memory thermoplastic polyurethane scaffolds triggered at body temperature for bone defect repair. Mater. Chem. Front..

[60] Zheng X., Zhang P., Fu Z., Meng S., Dai L., Yang H. (2021). Applications of nanomaterials in tissue engineering. RSC Adv..

[61] Lutzweiler G., Ndreu Halili A., Engin Vrana N. (2020). The Overview of Porous, Bioactive Scaffolds as Instructive Biomaterials for Tissue Regeneration and Their Clinical Translation. Pharmaceutics.

[62] Sultana N., Khan T.H. (2012). In Vitro Degradation of PHBV Scaffolds and nHA/PHBV Composite Scaffolds Containing Hydroxyapatite Nanoparticles for Bone Tissue Engineering. J. Nanomater..

[63] Divakar P., Moodie K.L., Demidenko E., Hoopes P.J., Wegst U.G. (2020). Quantitative evaluation of the in vivo biocompatibility and performance of freeze-cast tissue scaffolds. Biomed. Mater..

[64] Maleki A., Ghomi M., Nikfarjam N., Akbari M., Sharifi E., Shahbazi M.A., Kermanian M., Seyedhamzeh M., Nazarzadeh Zare E., Mehrali M. (2022). Biomedical Applications of MXene-Integrated Composites: Regenerative Medicine, Infection Therapy, Cancer Treatment, and Biosensing. Adv. Funct. Mater..

[65] Iravani S., Varma R.S. (2021). MXenes and MXene-based materials for tissue engineering and regenerative medicine: Recent advances. Mater. Adv..

[66] Hasan A., Morshed M., Memic A., Hassan S., Webster T.J., Marei H.E.S. (2018). Nanoparticles in tissue engineering: Applications, challenges, and prospects. Int. J. Nanomed..

[67] Khan I., Saeed K., Khan I. (2019). Nanoparticles: Properties, applications and toxicities. Arab. J. Chem..

[68] Joudeh N., Linke D. (2022). Nanoparticle classification, physicochemical properties, characterization, and applications: A comprehensive review for biologists. J. Nanobiotechnology.

[69] Khan Y., Sadia H., Shah S.Z.A., Khan M.N., Shah A.A., Ullah N., Ullah M.F., Bibi H., Bafakeeh O.T., Ben Khedher N. (2022). Classification, synthetic, and characterization approaches to nanoparticles, and their applications in various fields of nanotechnology: A review. Catalysts.

[70] Vasita R., Katti D.S. (2006). Nanofibers and their applications in tissue engineering. Int. J. Nanomed..

[71] Bramhill J., Ross S., Ross G. (2017). Bioactive nanocomposites for tissue repair and regeneration: A review. Int. J. Environ. Res. Public Health.

[72] Bao L., Cui X., Mortimer M., Wang X., Wu J., Chen C. (2023). The renaissance of one-dimensional carbon nanotubes in tissue engineering. Nano Today.

[73] Breuer T., Jimenez M., Humphrey J.D., Shinoka T., Breuer C.K. (2023). Tissue Engineering of Vascular Grafts: A Case Report From Bench to Bedside and Back. Arter. Thromb. Vasc. Biol..

[74] Antmen E., Vrana N.E., Hasirci V. (2021). The role of biomaterials and scaffolds in immune responses in regenerative medicine: Macrophage phenotype modulation by biomaterial properties and scaffold architectures. Biomater. Sci..

[75] Eltom A., Zhong G., Muhammad A. (2019). Scaffold techniques and designs in tissue engineering functions and purposes: A review. Adv. Mater. Sci. Eng..

[76] Al-Abduljabbar A., Farooq I. (2022). Electrospun polymer nanofibers: Processing, properties, and applications. Polymers.

[77] Shukla A., Dasgupta N., Ranjan S., Singh S., Chidambaram R. (2017). Nanotechnology towards prevention of anemia and osteoporosis: From concept to market. Biotech. Biotechnol. Equip..

[78] Sun J.L., Jiao K., Niu L.N., Jiao Y., Song Q., Shen L.J., Chen J.H. (2017). Intrafibrillar silicified collagen scaffold modulates monocytes to promote cell homing, angiogenesis, and bone regeneration. Biomaterials.

[79] Sultana N., Chang H.C., Jefferson S., Daniels D.E. (2020). Application of conductive poly(3,4-ethylenedioxythiophene): Poly(styrenesulfonate) (PEDOT:PSS) polymers in potential biomedical engineering. J. Pharm. Investig..

[80] Guo B., Lei B., Li P., Ma P.X. (2015). Functionalized scaffolds to enhance tissue regeneration. Regen. Biomater..

[81] Korniienko V., Husak Y., Diedkova K., Varava Y., Grebnevs V., Pogorielova O., Bērtiņš M., Korniienko V., Zandersone B., Ramanaviciene A. (2024). Antibacterial potential and biocompatibility of chitosan/polycaprolactone nanofibrous membranes incorporated with silver nanoparticles. Polymers.

[82] Wang Y., Han X., Li J., Xu Y., Sun Y., Zhang X. (2023). Intelligent vascularized 3D/4D/5D/6D-printed tissue scaffolds for bone tissue repair: Tuning mechanical structures and biological properties. Nano-Micro Lett..

[83] Singh A.V., Bhardwaj P., Upadhyay A.K., Pagani A., Upadhyay J., Bhadra J., Tisato V., Thakur M., Gemmati D., Mishra R. (2024). Navigating regulatory challenges in molecularly tailored nanomedicine. Explor. BioMat-X.

